# The Role and Mechanism of Incretins in Gynaecologic Diseases

**DOI:** 10.1002/edm2.70340

**Published:** 2026-09-25

**Authors:** Jiayu Yan, Minyue Cao, Yan Ding, Yiqin Zhang, Yihan Sun, Genyi Jiang, Yanli Zhang, Luyao Kang, Xue Zhou, Jing Luo, Bilan Li

**Affiliations:** ^1^ Shanghai Key Laboratory of Maternal Fetal Medicine, Shanghai Institute of Maternal‐Fetal Medicine and Gynecologic Oncology, Shanghai First Maternity and Infant Hospital, School of Medicine Tongji University Shanghai China

**Keywords:** GLP‐1, gynaecological cancers, incretins, insulin resistance, polycystic ovary syndrome

## Abstract

**Background:**

Obesity is a major global public health concern closely linked to the development and progression of various gynaecological disorders, particularly polycystic ovary syndrome (PCOS) and endometrial cancer. Incretins, a group of gut‐derived hormones primarily including glucagon‐like peptide‐1 (GLP‐1) and glucose‐dependent insulinotropic polypeptide (GIP), are widely recognised for their roles in glycemic control and insulin secretion. Accumulating evidence reveals that GLP‐1 and GIP also participate in modulating inflammation, autophagy, immune response and gut‐brain communication through multiple signalling pathways, thereby playing important roles in female reproductive disorders. This review provides a comprehensive overview of the mechanisms and therapeutic potential of incretins in gynaecological conditions.

**Methods:**

We performed a comprehensive literature search across PubMed, Web of Science and Embase, covering studies published from January 1995 to July 2026. Search terms combined incretin‐related keywords (GLP‐1, GIP, GLP‐1 receptor agonists) with gynaecological disease terms (polycystic ovary syndrome, endometriosis, endometrial cancer, ovarian cancer, intrauterine adhesion). We primarily included original research studies and authoritative reviews, and excluded meeting abstracts, case reports and non‐English literature.

**Results:**

GLP‐1 receptor agonists (GLP‐1 RAs) exert multi‐faceted benefits in PCOS, endometriosis, intrauterine adhesions and gynaecologic cancers through cAMP/PKA, PI3K and AMPK signalling pathways. Special emphasis is placed on the multi‐faceted benefits of GLP‐1 RAs, while evidence supporting GIP‐based therapies in gynaecology remains relatively limited and largely preclinical. The current status of clinical application, safety considerations and future directions are further discussed.

**Conclusion:**

Incretin‐based therapies offer novel approaches for metabolic and oncological gynaecological diseases, with significant translational value. Large‐scale clinical studies and deeper mechanistic exploration are warranted to realise their full therapeutic potential.

## Graphical abstract

This review outlines the mechanisms of incretins in polycystic ovary syndrome, endometriosis and gynaecological cancers, highlighting the translational potential of incretin‐based therapies for metabolic‐associated gynaecological disorders.

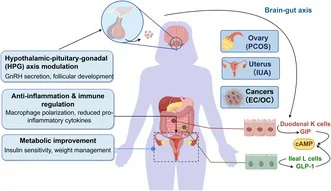

## 1 Introduction

Obesity has been recognised as a major global public health challenge for decades. In 1997, the World Health Organization formally classified obesity as a chronic disease, marking a turning point in global recognition and research on this condition. According to the World Obesity Atlas 2025, the number of obese adults worldwide is projected to rise from 810 million in 2020 to 1.53 billion in 2035 [1]. In recent years, accumulating evidence on obesity and tumour biology has demonstrated that tumour immunity is modulated by metabolic disorders including obesity and diabetes. Among the 13 types of obesity‐related cancers, ovarian and endometrial cancers are of particular relevance to women's health [2]. Meanwhile, the rising prevalence of obesity and diabetes among adolescents disproportionately affects girls, with long‐term impacts on reproductive health [3]. Also for women, the impact of obesity in female reproduction is of greater concern and its relationship to endocrinology, metabolism and infertility may be very strong [4, 5]. One of the more studied existing syndromes is PCOS, whose main pathophysiological features are hyperandrogenemia and insulin resistance (IR), often accompanied by overweight or obesity and abnormalities of glucolipid metabolism [6]. With intensive research on obesity and diabetes treatments, enteroendocrine hormones and brain‐gut axis‐mediated hormonal regulation have received growing attention. Incretins, mainly including GLP‐1 and GIP, represent a class of intestinal hormones with pleiotropic biological effects. The clinical development of incretin‐based drugs has opened new possibilities for the management of PCOS and other gynaecological disorders, yet the underlying mechanisms remain incompletely understood. This review systematically summarises the biological characteristics of incretins, their mechanisms of action in gynaecological diseases and their clinical translational potential.

This article is a narrative review. We performed a comprehensive literature search across PubMed, Web of Science and Embase, covering studies published from January 1995 to July 2026. Search terms combined incretin‐related keywords (GLP‐1, GIP, GLP‐1 receptor agonists) with gynaecological disease terms (polycystic ovary syndrome, endometriosis, endometrial cancer, ovarian cancer, intrauterine adhesion). We primarily included original research studies and authoritative reviews and excluded meeting abstracts, case reports and non‐English literature.

## 2 Incretins

**FIGURE 1:**
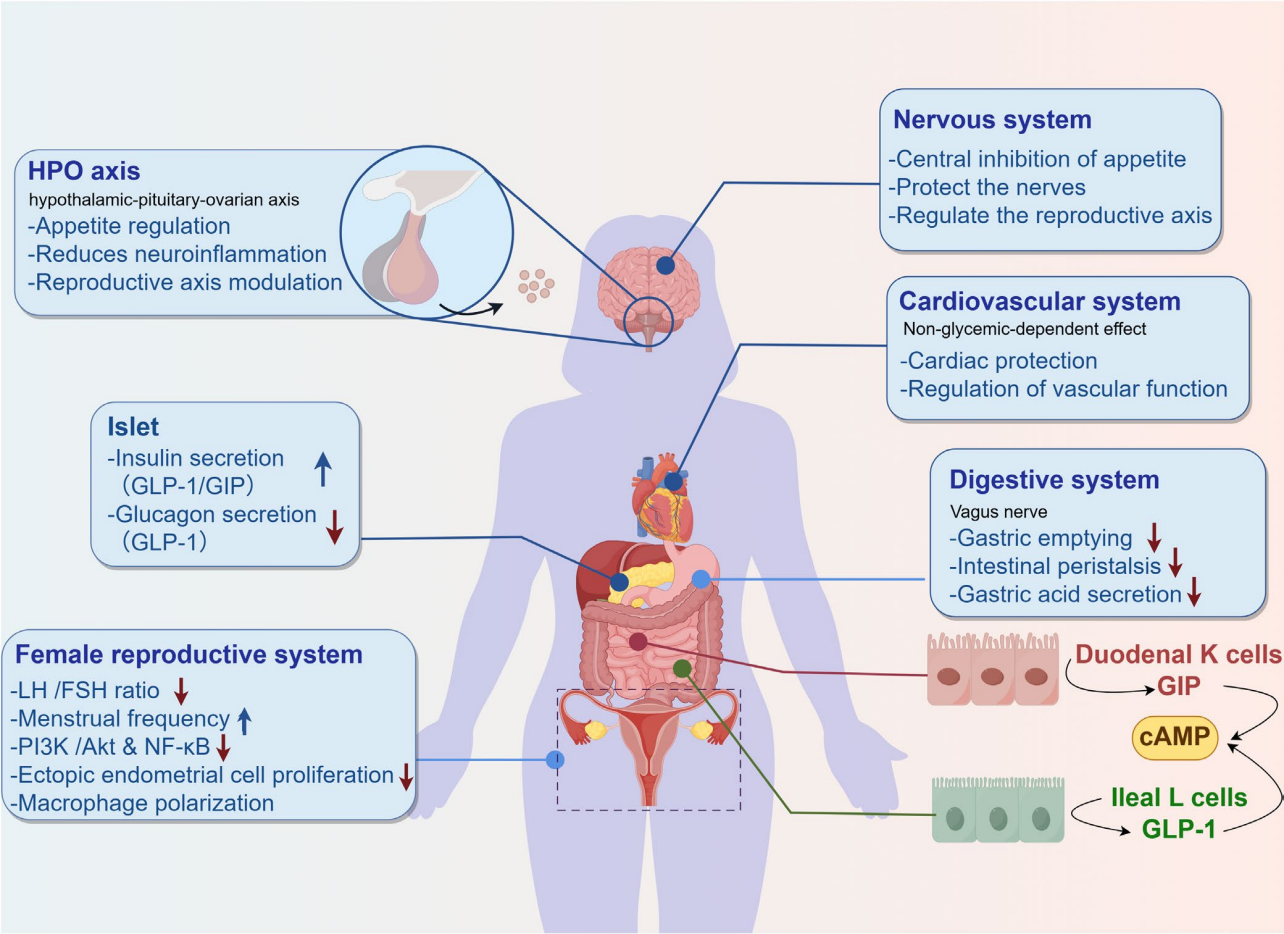
The role of incretins in various systems of women.

Incretins are intestinal‐derived hormones that play a core role in the entero‐insular axis, regulating postprandial insulin secretion in a glucose‐dependent manner. The two main characterised incretins are GLP‐1 and GIP. Beyond their classical glycemic effects, incretins exert extensive extra‐pancreatic effects across multiple systems, as summarised in Figure 1.

### 2.1 GLP‐1

GLP‐1, a 30‐amino acid polypeptide, is predominantly secreted by L cells located in the terminal ileum and colon, with primary expression in the intestinal and hypothalamic systems [7, 8]. The GLP‐1 receptor (GLP‐1R), a member of the G protein‐coupled receptor family, is widely distributed across pancreatic islets, adipose tissue, cardiovascular system, central nervous system and reproductive organs, mediating diverse biological effects [9]. For example, GLP‐1 has significant effects on insulin resistance, vasodilation, cardiac effects, metabolic effects and blood glucose and insulin secretion [10, 11]. The classical mechanism of GLP‐1 involves activation of the cAMP/PKA signalling cascade upon binding to β‐cell receptors, enhancing insulin gene transcription and glucose‐dependent insulin secretion [12]. Liu et al. identified a functional GLP‐1R antibody that modulates GLP‐1R signalling through PKA‐mediated phosphorylation of Raptor at Ser791 [13]. Le et al. further demonstrated in Chinese hamster ovary cells that GLP‐1R activation stimulates mTORC1 activity via PKA‐dependent Raptor phosphorylation [14]. GLP‐1 may also regulate voltage‐dependent Ca^2+^ channels through the cAMP pathway, thereby promoting Ca^2+^ influx to stimulate insulin release [15]. Additionally, on the other hand, GLP‐1 acts on receptors in pancreatic α cells, reducing the secretion of glucagon. Farhy et al., using mathematical modelling, found that GLP‐1 affects glucose homeostasis by inhibiting glucagon secretion from pancreatic alpha cells while promoting insulin secretion [16].

GLP‐1 also participates in the phosphatidylinositol 3‐kinase (PI3K) signalling pathway, promoting insulin secretion and enhancing peripheral insulin sensitivity. Zhang et al. found that progesterone receptor membrane component 1 (PGRMC1) interacts with GLP‐1R, strengthening the cAMP/EPAC pathway and activating epidermal growth factor receptor (EGFR) and its downstream PI3K cascade, leading to increased calcium influx and insulin exocytosis [17]. Furthermore, GLP‐1 promotes β‐cell proliferation and differentiation via the PI3K/PDX‐1 pathway, upregulating genes related to glucose transport and metabolism [18].

In recent years, novel downstream mediators of GLP‐1 action have been identified. Zinc‐α2‐glycoprotein (ZAG) is a novel adipokine associated with insulin sensitivity. Adipose ZAG expression is decreased in patients with Type 2 diabetes mellitus and 12‐week liraglutide treatment significantly elevates circulating ZAG levels. These findings suggest that ZAG may act as a positive regulator of insulin sensitivity and mediate the metabolic benefits of GLP‐1 RAs, though its exact role in IR pathogenesis requires further investigation [19].

A growing body of research is uncovering new mechanisms for upregulating GLP‐1 secretion through the Takeda G protein‐coupled receptor 5 (TGR5) and GPR119 signalling pathways. Wang et al. have identified Imperatorin, a dietary compound, as an activator of TGR5 and GPR119 receptors, which enhances the levels of GLP‐1 in plasma of a type 1 diabetic rat model. The study demonstrated that Imperatorin dose‐dependently increases TGR5‐mediated glucose uptake in Chinese hamster ovary cells (CHO‐K1 cells) and induces GLP‐1 secretion in NCI‐H716 cells in vitro [20]. Wang et al. conducted experiments in streptozotocin (STZ)‐induced Type 1 diabetic rat models and discovered that glycyrrhizic acid (GA), a principal component of licorice, can increase the secretion of GLP‐1 by activating the TGR5. In vitro studies with CHO‐K1 cells transfected with the TGR5 gene showed that GA directly targeted TGR5, enhancing glucose uptake and cyclic AMP (cAMP) levels. Additionally, in GLP‐1‐expressing intestinal NCI‐H716 cells, GA promoted GLP‐1 secretion along with a significant increase in calcium levels [21].

Harikumar et al.'s study, utilising recombinant expression and bioluminescence resonance energy transfer (BRET) technology, revealed that the GLP‐1R and secretin receptor (SecR) can form heterodimers when co‐expressed in islet cells, primarily dependent on the lipid face of transmembrane segment 4 (TM4). These heterodimers reduce the endogenous calcium response to secretin stimulation but have no effect on the cAMP response. Additionally, they mediate agonist‐induced cross‐desensitisation of the receptors, suggesting that further exploration of novel molecular mechanisms is warranted in naturally co‐expressing receptor settings [22].

Beyond pancreatic effects, GLP‐1 slows gastric emptying via gastrointestinal neuroendocrine cells and the vagus nerve and suppresses appetite by activating hypothalamic GLP‐1 receptors and modulating neuropeptide Y and pro‐opiomelanocortin expression [23]. GLP‐1 also exerts cardiovascular protective effects, including improving endothelial function, lowering blood pressure and reducing atherosclerosis, partially mediated by the NO/cGMP pathway and endothelial nitric oxide synthase activation [9, 24, 25]. These multi‐system effects lay the foundation for the therapeutic potential of GLP‐1 in gynaecological diseases.

### 2.2 GIP

GIP, a 42‐amino acid peptide, is primarily produced by K cells in the duodenum and jejunum [26]. The GIP receptor (GIPR) is expressed in various tissues throughout the body, with physiological functions that include increasing insulin biosynthesis, promoting pancreatic β‐cell differentiation and reducing apoptosis, as well as enhancing adipose tissue accumulation and bone formation [27]. Research on GIP is less extensive compared to that on GLP‐1.

Kubota et al. discovered that GIP activates the cAMP signalling pathway through its receptor, thereby influencing insulin secretion. They also investigated two common mutations: the Gly198Cys mutation significantly reduced the sensitivity of GIPR, while the Glu354Gln mutation had no significant impact on GIPR function [28]. Wheeler et al. cloned rat islet GIPR complementary DNA to study the ligand binding and intracellular signalling characteristics of GIPR. They found that GIP activates adenylate cyclase and the cAMP signalling pathway through its receptor, GIP‐R1, thereby enhancing insulin secretion [29]. Ehses et al. found that GIP promotes the release of arachidonic acid (AA) by activating adenylate cyclase (AC) and increasing intracellular cyclic adenosine monophosphate (cAMP) levels in Chinese hamster ovary K1 cells and a β‐cell model (βTC‐3). This process is independent of extracellular calcium ions and is mediated by the cAMP signalling pathway [30].

The action of GIP also requires the involvement of GLP‐1. Samms et al.'s research, through analysing the role of GIP in regulating adipose tissue function and energy balance, discovered that GIP enhances lipoprotein lipase (LPL) activity and promotes fat storage in adipocytes through its receptor GIPR, thereby increasing insulin sensitivity. This may be achieved by modulating the recruitment and activity of LPL through the PI3K/PKB‐LKB1/AMPK‐TORC2 signalling axis, which facilitates the uptake and storage of lipids in adipose tissue [31]. Whitaker et al.'s study revealed that N‐glycosylation sites of GIPR are glycosylated when expressed in vitro, reducing the degradation of GIPR in the endoplasmic reticulum and promoting its transport from the endoplasmic reticulum to the cell membrane. Furthermore, the function of N‐glycosylation‐deficient GIPR can be rescued by co‐expression with wild‐type GLP‐1R, suggesting that they may form a functional heterodimer [32].

**FIGURE 2:**
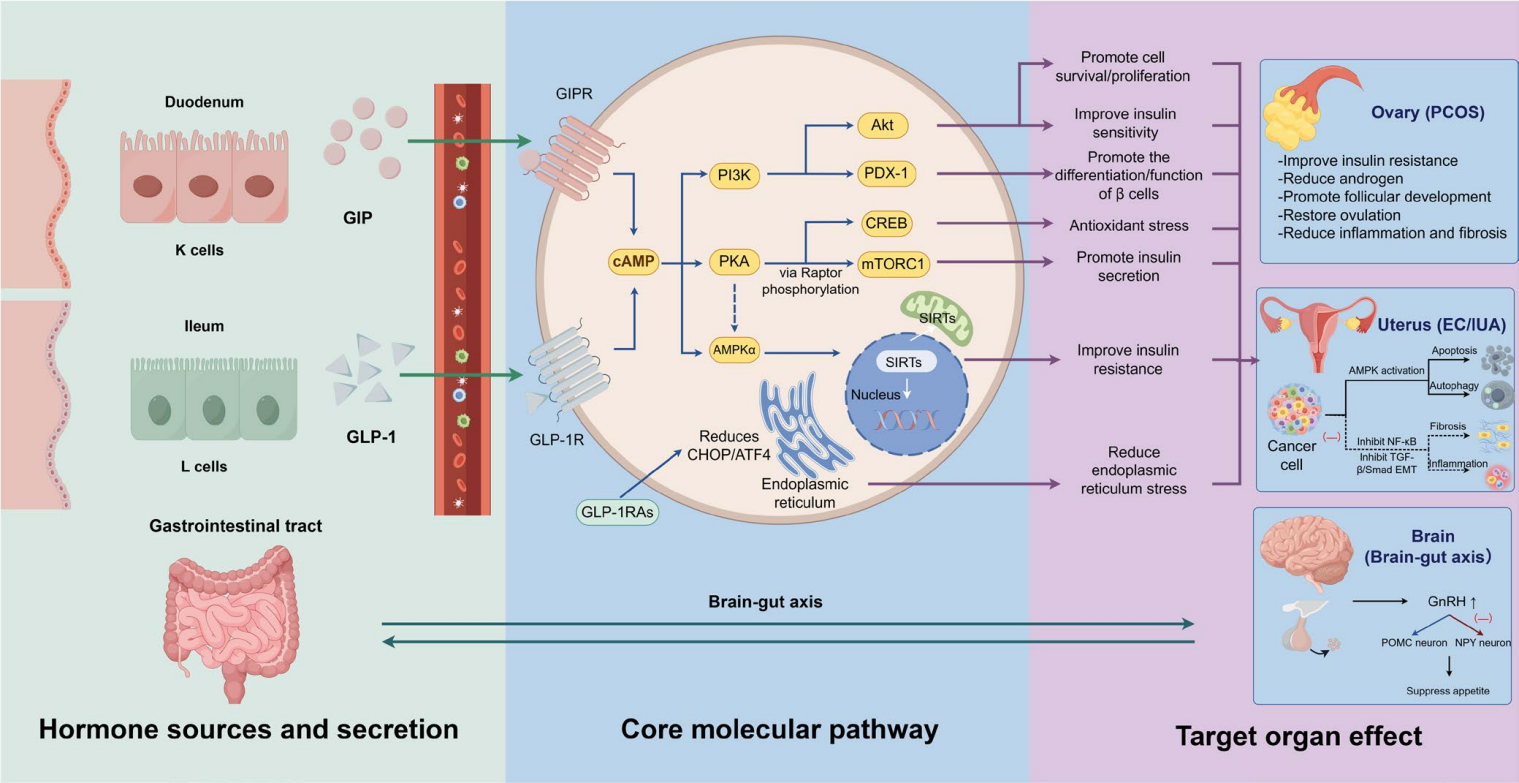
The pathways and functions of incretins.

Taken together, incretins act through complex and interconnected signalling networks to regulate metabolism, inflammation and endocrine function. The core pathways and biological functions are schematically summarised in Figure 2.

## 3 The Role of Incretin in the Treatment of Gynaecological Diseases

The rising prevalence of metabolic disorders has highlighted the close link between metabolic dysregulation and gynaecological diseases. Incretin‐based therapies, originally developed for diabetes and obesity, have shown promising therapeutic potential in various gynaecological conditions. It should be noted that at present, incretin analogs are not first‐line targeted therapies for gynaecological diseases, but serve as potential adjuvant treatment strategies, especially for patients with metabolic comorbidities.

### 3.1 Fertility Function and Ovarian Diseases

In the context of women's ovarian and reproductive health, it is evident that the incidence of related diseases is becoming increasingly younger and more widespread, causing numerous disturbances in women's lives. Among these, the most relevant to incretins is PCOS [33, 34, 35]. Studies have confirmed abnormal secretion of GIP and GLP‐1 in patients with PCOS [36]. Etrusco et al. found that GLP‐1RAs such as liraglutide and semaglutide can improve menstrual regularity, ovulation rates and reduce body fat in patients with PCOS. Furthermore, the therapeutic applications of GLP‐1RAs in cardiovascular health (inhibiting the proliferation and migration of vascular smooth muscle cells, as well as vascular calcification), kidney function, brain‐gut axis and neuronal health and the immune system (reducing the expression of inflammatory factors such as TNF‐α and IL‐6) will be detailed in subsequent sections [37, 38, 39].

Therapies targeting the incretin pathway for the treatment of PCOS have been a focus of researchers in recent years. Identifying more stable and efficient mechanisms within this pathway is crucial. Bednarz et al. found that GLP‐1RAs enhance the antioxidant capacity of pancreatic beta cells by activating the cAMP‐PKA‐CREB signalling pathway and improve oxidative stress through the Nrf2/ARE pathway, thereby protecting the function of pancreatic beta cells [40]. Additionally, GLP‐1RAs increase intracellular Ca^2+^ concentration by raising cAMP levels, stimulating the secretion of insulin‐containing vesicles and promoting the release of insulin into the bloodstream. As mentioned earlier, they also activate the epidermal growth factor receptor to promote PI3K synthesis and DNA replication, enhancing beta cell proliferation. Furthermore, GLP‐1RAs modulate the protein kinase R‐like endoplasmic reticulum (PERK) pathway, targeting the expression of activating transcription factor 4 (ATF4) and C/EBP homologous protein (CHOP), reducing endoplasmic reticulum stress and improving insulin resistance in adipose tissue [37].

Tao et al. identified that the GLP‐1 receptor agonist exenatide improves insulin resistance in PCOS rats by activating the AMPKα‐SIRT1 signalling pathway, with significant increases in both protein and mRNA expression levels of AMPKα and SIRT1 observed after treatment [41]. Genetic evidence has confirmed that genetic activation of GIPR is associated with a reduced risk of PCOS, and GIP signalling is involved in the regulation of estrous cycle and female fertility by affecting pituitary‐gonadal axis function. Zhang et al., using a two‐sample Mendelian Randomisation (MR) analysis approach, found that genetic activation of GIPR is associated with a reduced risk of PCOS, providing genetic evidence for this association [42]. Svendsen et al. discovered that metformin enhances the incretin effect in women with PCOS by increasing levels of GIP and GLP‐1 [43].

The farnesoid X receptor (FXR) pathway represents an important link between gut microbiota, GLP‐1 secretion and ovarian dysfunction. Gut microbiota dysbiosis in PCOS leads to elevated microbial metabolite agmatine, which activates FXR and suppresses GLP‐1 secretion from intestinal L cells, contributing to insulin resistance and ovarian dysfunction [44, 45]. This agmatine‐FXR‐GLP‐1 signalling axis plays a significant role in PCOS pathogenesis [46].

Mokou et al. found that serum levels of Fetuin‐B are significantly higher in women with PCOS compared to healthy females, and this increase is associated with insulin resistance (IR). This suggests that GLP‐1RAs may play a role in the treatment of PCOS by enhancing insulin sensitivity and reducing circulating Fetuin‐B levels associated with IR [47].

Incretin receptors have been shown to act in multiple systems, indicating that the GLP‐1/GIP pathway can also ameliorate complications across various systems. GLP‐1RAs possess cardioprotective and renoprotective effects, and they have the potential to be used in combination with renin‐angiotensin‐aldosterone system inhibitors for the treatment of diabetic nephropathy [48], reducing the risk of cardiovascular disease (CVD) and improving non‐alcoholic fatty liver disease [49]. Liraglutide significantly improves food intake, body weight, fat mass and insulin resistance index in PCOS rats, and also ameliorates dyslipidemia and leptin levels in these rats. It has a positive impact on cardiovascular and metabolic risk factors in postmenopausal PCOS rats, although its blood pressure‐lowering effects are influenced by hyperandrogenemia [50].

### 3.2 Inflammation, Immunity and Gynaecological Tumours

Numerous studies have found that GLP‐1 can inhibit inflammation mainly by reducing the secretion of macrophage inflammatory factors (such as IL1β, IL6 and TNF‐β) [51, 52]. Lee et al. conducted a study using recombinant adenovirus (rAd)‐mediated GLP‐1 gene therapy in an obese diabetic mouse model. They observed that GLP‐1 could reduce the infiltration of macrophages in adipose tissue and decrease the expression of genes specific to M1‐type macrophages. Additionally, GLP‐1 reduced the expression and production of inflammatory factors such as IL‐6, TNF‐α and MCP‐1. Concurrently, GLP‐1 also inhibited the activation of NF‐κB and JNK signalling pathways associated with inflammatory responses [53]. Jiang et al. observed in mouse models of obesity and insulin resistance that GLP‐1 reduced the expression of CHOP in adipocytes under endoplasmic reticulum (ER) stress and increased insulin‐stimulated AKT phosphorylation (p‐AKT). GLP‐1 modulates the ER stress response by enhancing autophagosome formation and increasing the ratio of LC3II to LC3I, thereby inhibiting mTOR activity [54]. Recent groundbreaking research by Wong et al. has identified that the anti‐inflammatory effects of GLP‐1RAs require the involvement of central nervous system GLP‐1Rs. These effects can be mediated through two distinct pathways: the central adrenergic and opioid G protein‐coupled receptors (GPCRs), both of which can reduce the production of tumour necrosis factor α (TNF‐α) to suppress peripheral inflammation [55].

Wu et al. further discovered that Semaglutide alleviates fibrosis and inflammation in an intrauterine adhesion (IUA) model by modulating the NF‐κB signalling pathway. It reduces the mRNA levels of fibrotic markers ACTA2, COL1A1 and FN induced by TGF‐β1 in human mesenchymal cells, as well as the expression of inflammatory markers TNF‐α, IL‐6 and NF‐κB. Additionally, it inhibits epithelial‐mesenchymal transition (EMT) by affecting the expression of TGF‐β, TGF‐β receptors and Snail1 [56]. In a mouse model of IUA induced by mechanical scraping and inflammation, Dulaglutide was found to improve uterine morphology, increase endometrial thickness and gland number and significantly reduce collagen fibre deposition in the endometrium by suppressing M1 macrophage polarisation and the release of inflammatory factors. It may also ameliorate fibrosis and inhibit EMT by inhibiting the TGF‐β/Smad2 signalling pathway [57].

In addition to endometrial cancer and IUA, incretins also show potential therapeutic value in endometriosis. Preclinical studies have confirmed that GLP‐1RAs can inhibit the proliferation of ectopic endometrial cells through PI3K/Akt and NF‐κB pathways, regulate macrophage polarisation, reduce the secretion of pro‐inflammatory factors such as IL‐6 and TNF‐α and alleviate the progression of endometriosis in animal models.

GLP‐1 RAs have been shown to inhibit tumour cell growth, promote apoptosis and enhance chemotherapy sensitivity, particularly in pancreatic, breast and endometrial cancers [58]. Specifically, GLP‐1 RAs can suppress the proliferation of endometrial cancer cells through the AMPK signalling pathway [59, 60]. Devis‐Jauregui et al. investigated the effects of the GLP‐1 analog liraglutide on autophagy in endometrial cancer cells and found that liraglutide induces autophagy and cell death by increasing LC3 expression and p‐AMPKα, while decreasing SQSTM1 protein levels. They also discovered that the combination of GLP‐1R agonists with the AMPK activator AICAR enhances the accumulation of autophagosomes and early apoptosis rates, suggesting that GLP‐1 signalling may influence the progression of endometrial cancer by modulating autophagy [61]. In a study by Zhu et al., liraglutide was found to significantly inhibit the proliferation of endometrial cancer cells (IK and HEC‐1B cell lines) in a time‐ and concentration‐dependent manner. It was observed that liraglutide activates the AMP‐activated protein kinase (AMPK) signalling pathway, upregulates the expression of progesterone receptors (PGR) and shows a synergistic inhibitory effect on endometrial cancer cell proliferation when used in combination with medroxyprogesterone acetate (MPA) [62].

For ovarian cancer, preclinical studies have found GLP‐1R expression in some ovarian cancer tissues, and GLP‐1 RAs can inhibit ovarian cancer cell proliferation and invasion via the AMPK pathway. However, research in this area is still in its early stages and the clinical translational value remains to be explored.

It should be emphasised that the anti‐tumour effects observed in preclinical studies may be partially secondary to weight loss and improved metabolic status. The direct anti‐tumour effects of incretins on gynaecologic cancers require further validation in clinical studies.

### 3.3 Brain‐Gut Axis and Endocrine

Accumulating evidence suggests that incretins can directly modulate the hypothalamic—pituitary—adrenal (HPA) and hypothalamic—pituitary‐gonadal (HPG) axes, providing an endocrine mechanism for their effects on reproductive health [63, 64]. Gastrointestinal microbiota dysbiosis, commonly observed in women with PCOS, can impact female reproductive function through the brain‐gut axis. The gut microbiota–incretin–reproductive axis forms a complex regulatory network: gut microbiota dysbiosis impairs GLP‐1 secretion from intestinal L cells, which in turn disrupts hypothalamic GnRH secretion and HPG axis function, contributing to reproductive and metabolic disorders in PCOS [65, 66]. Comninos et al. have analysed the interplay between gastrointestinal hormones and the reproductive axis, revealing the significant role of gastrointestinal hormones, such as GLP‐1 and GIP, in the secretion of gonadotropin‐releasing hormone (GnRH) in the hypothalamus [67, 68]. GLP‐1 Ras modulate the release of GnRH by activating GLP‐1 receptors in the hypothalamic—pituitary‐gonadal axis, thereby affecting the secretion of insulin and glucagon, with dense expression of GLP‐1 mRNAs detectable in the central nervous system [69]. Farkas et al., through electrophysiological recordings and immunohistochemical methods, discovered that GLP‐1 directly acts on GnRH neurons in male mice via its receptor (GLP‐1R), increasing the firing rate of these neurons and the frequency of GABAergic miniature postsynaptic currents (mPSCs), suggesting that GLP‐1 regulates GnRH release and impacts nitric oxide (NO) and endocannabinoid pathways [70]. Additionally, GLP‐1 increases the electrical activity of hypothalamic POMC neurons, activating them in the lateral hypothalamus of rats through PKA and L‐type calcium channels, thereby reducing feeding behaviour [71]. Abdalla et al. analysed that GLP‐1 may improve ovarian morphology and gonadotropin levels by modulating steroid hormone levels and improving metabolic health, potentially influencing the brain‐gut axis. Furthermore, Tao et al., using animal models, observed that GLP‐1 RAs can alleviate diabetes‐induced ovarian inflammation, fibrosis, oxidative stress and increase anti‐Müllerian hormone (AMH) levels [72].

GLP‐1RAs have been found to have direct regulatory effects on the hypothalamic–pituitary–gonadal— (HPG) axis. Beyond their metabolic and glucose‐related regulatory functions, Khan et al.'s study, which involved the deletion of the GIPR in a mouse model, revealed that GIPR knockout mice exhibited significant disruptions in estrous cycles and a decrease in fertility, suggesting that GIP signalling may modulate female reproductive functions by affecting the function of the pituitary‐gonadal axis [73]. GIPR knockout in mice can lead to significant estrous cycle disorder and decreased fertility, suggesting that GIP plays an irreplaceable role in maintaining normal female reproductive function. Research has shown that GLP‐1RAs may increase the pulsatile release of gonadotropin‐releasing hormone (GnRH) by activating the hypothalamic kisspeptin system, thereby influencing the secretion of luteinising hormone (LH) [74]. Additionally, at the peripheral level, GLP‐1RAs may promote follicular development and oocyte maturation by regulating the proliferation and anti‐apoptotic effects of theca cells [75].

Notably, Sánchez‐Garrido et al. applied a dual GLP‐1/oestrogen agonist (GLP‐1/E) in two PCOS mouse models and found that it significantly improved ovarian cyclicity and insulin sensitivity by regulating the HPG axis, without direct estrogenic proliferative effects on the uterus. GLP‐1/E also exerted anti‐inflammatory and anti‐fibrotic effects in ovarian tissue, particularly in obesity‐ and diabetes‐related reproductive damage. Additionally, in diabetic mouse models, GLP‐1/E reduces body weight and ameliorates glucotoxicity, which in turn improves sperm quality [76, 77].

Moreover, studies have found that the Notch signalling pathway is overexpressed in PCOS rats, leading to Aβ aggregation, cell apoptosis and neuronal damage, which also affects the rats’ learning and memory abilities. However, liraglutide can repair memory impairment by modulating downstream targets of the Notch signalling pathway, while also reducing Aβ aggregation and cell apoptosis. This suggests that cognitive disorders and neurological diseases may also be linked to the brain‐gut axis [78].

## 4 Current Status of Clinical Application of Incretin

An increasing number of studies have confirmed that the combination of GLP‐1RAs with medications such as metformin can effectively reduce body weight, BMI and waist circumference, as well as improve insulin sensitivity [79, 80], showing a significant enhancement in therapeutic outcomes compared to metformin monotherapy [81, 82]. The efficacy and safety of monotherapy in reducing weight and androgen levels, as well as improving cardiometabolic profiles in women with PCOS and obesity, have been clinically confirmed [83]. Additionally, a growing number of combination therapies are under continuous investigation, such as the significant effectiveness of linagliptin and/or indole‐3‐carbinol (I3C) in treating PCOS [84]. In a randomised single‐blind trial conducted by Jensterle et al., semaglutide treatment was found to significantly reduce lingual fat tissue and adipose proportion in patients with PCOS compared to placebo [85]. Furthermore, clinical studies have confirmed that treatment with GLP‐1RAs followed by a two‐year discontinuation still results in a significant reduction in weight and metabolic markers [86]. However, some studies indicate that combination therapies may lead to a higher incidence of adverse drug events compared to metformin monotherapy [87, 88], highlighting the need for extensive research on the safety profile of GLP‐1RAs.

In addition to the six currently approved and utilised GLP‐1RAs, ongoing research is exploring a plethora of additional pharmaceuticals. Wang et al. have identified a novel recombinant dual agonist, DR10601, which exhibits dual agonistic activity on both GLP‐1 and GIP receptors [89]. Wooten et al. have elucidated the regulatory mechanisms of flavonoid compounds on the GLP‐1R signalling pathway, providing a structural foundation for the development of new small molecule GLP‐1R modulators [90]. Gasbjerg et al. have, for the first time, validated the efficacy of GIP(3–30)NH2 as a GIP receptor antagonist in humans, demonstrating that GIP(3–30)NH2 can significantly reduce GIP‐induced insulin secretion by 82% and lower the required glucose levels to placebo levels [91]. Furthermore, selective agonists for GPR120 have been found to enhance the release of GLP‐1 and glucose‐stimulated insulin secretion (GSIS), as well as inhibit LPS‐induced macrophage inflammatory responses [92, 93].

Notably, tirzepatide, the first approved dual GIP/GLP‐1 receptor agonist, has demonstrated superior weight loss and glycemic control compared with selective GLP‐1 RAs in patients with Type 2 diabetes and obesity. Preliminary exploratory studies suggest its potential benefits in improving reproductive and metabolic parameters in women with PCOS [94, 95], but large‐scale clinical trials in gynaecological populations are still ongoing [96].

## 5 Limitations and Safety Considerations

### 5.1 Limitations of Current Evidence

Despite the encouraging signals emerging from preclinical models and early‐phase clinical studies, several limitations inevitably temper the strength of the conclusions that can be drawn. A central difficulty remains the disentanglement of direct, receptor‐mediated actions of incretins on reproductive tissues from the indirect benefits that arise through weight loss and the amelioration of systemic insulin resistance. Although GLP‐1 receptor expression has been documented throughout the female reproductive tract—including the ovary and endometrium—and GLP‐1R knockout mice display delayed puberty and reduced ovarian follicle counts, observations that strongly suggest direct gonadal actions, the majority of clinical improvements in menstrual regularity and ovulation have been documented in parallel with substantial weight reduction [97, 98, 99]. As Baggio and Drucker have previously cautioned for other peripheral tissues, some of the metabolic actions attributed to GLP‐1 may in fact be indirect, a caveat that applies with equal force to reproductive endpoints [100]. Well‐designed tissue‐specific knockout models and adequately powered clinical studies that rigorously control for the degree of weight loss are therefore urgently needed to resolve this ambiguity. Beyond polycystic ovary syndrome, the evidence base remains conspicuously immature. As illustrated in Table 1, clinical data for conditions such as endometriosis, intrauterine adhesions and gynaecological malignancies are scant, with most mechanistic insights still derived from in vitro assays or animal models [97, 101, 102]. A 2025 umbrella review noted that the understanding of incretin action in human ovarian tissue is ‘almost exclusively’ extrapolated from non‐human systems, and no large‐scale randomised controlled trial has yet evaluated a GLP‐1 receptor agonist for a primary reproductive endpoint in any non‐PCOS gynaecological disorder [102]. The therapeutic promise in these indications therefore remains hypothetical until confirmed by dedicated clinical investigation. Adding to the uncertainty, treatment response is notably heterogeneous. Real‐world data from a cohort of 483 adults prescribed semaglutide or liraglutide for obesity classified 17.8% as non‐responders (< 5% total body weight loss at a mean follow‐up of 520 days), while only one‐third achieved a hyper‐response (> 15% loss) [103]. Predictive factors for response in gynaecological populations—such as baseline endocrine profile, body composition or genetic background—have not been systematically characterised, and the mechanisms underlying this inter‐individual variability remain poorly understood [104]. Compounding these concerns, the existing clinical literature suffers from significant methodological constraints. Most published trials are limited by small sample sizes, short follow‐up durations and a reliance on intermediate metabolic surrogates—including HOMA‐IR, androgen levels and cycle regularity—rather than on patient‐centred reproductive outcomes such as pregnancy rate, live birth rate or time‐to‐conception [97, 101, 105]. A 2022 systematic review specifically highlighted the absence of a control arm in some studies and the lack of large, well‐organised, double‐blind, placebo‐controlled studies over longer periods as critical gaps [105]. Collectively, these design shortcomings preclude robust conclusions about the long‐term efficacy and safety of incretin‐based therapies for fertility indications, underscoring the need for more rigorous, outcome‐driven clinical research in this field.

**TABLE 1:** Summary of evidence for incretin‐based therapies in gynaecological diseases.

Disease entity	Intervention category	Study type & evidence base	Key findings	Translational stage	Core limitations
Polycystic ovary syndrome (PCOS)	GLP‐1 receptor agonists (monotherapy or combined with metformin)	Randomised controlled trials (RCTs), meta‐analyses, prospective cohort studies	Improves menstrual regularity, ovulation rate, body composition, insulin resistance and hyperandrogenism; combination therapy yields greater metabolic benefits than metformin alone	Moderate clinical evidence (off‐label use)	Most studies have small sample sizes and short follow‐up; data on long‐term reproductive outcomes (pregnancy, live birth) are insufficient
PCOS	GIP pathway modulation	Mendelian randomisation studies, animal experiments, mechanistic studies	Genetic activation of GIPR is associated with reduced PCOS risk; GIP signalling regulates pituitary‐gonadal axis function	Early exploratory stage	No clinical interventional studies; causal relationship in humans remains to be validated
Endometriosis	GLP‐1 receptor agonists	In vitro cell experiments, rodent model studies	Inhibits proliferation of ectopic endometrial cells via PI3K/Akt and NF‐κB pathways; regulates macrophage polarisation and reduces pro‐inflammatory cytokine secretion	Preclinical evidence dominant	Lack of clinical trial data; it remains unclear whether benefits are independent of weight loss
Intrauterine adhesion (IUA)	GLP‐1 receptor agonists (semaglutide, dulaglutide)	In vitro cell experiments, mouse model studies	Ameliorates endometrial fibrosis and inflammation via NF‐κB and TGF‐β/Smad pathways; inhibits epithelial‐mesenchymal transition	Preclinical evidence dominant	Only animal and cellular evidence; no human clinical data
Endometrial cancer	GLP‐1 receptor agonists	In vitro cell line experiments, xenograft model studies	Inhibits cell proliferation, induces autophagy and apoptosis via AMPK pathway; upregulates progesterone receptor expression and shows synergism with progestin therapy	Preclinical evidence dominant	Lack of clinical cohort and interventional evidence; direct anti‐tumour effect vs. indirect metabolic effect cannot be distinguished
Ovarian cancer	GLP‐1 receptor agonists	In vitro cell experiments, preliminary observational studies	GLP‐1R is expressed in partial ovarian cancer tissues; inhibits cell proliferation and invasion via AMPK pathway	Early exploratory stage	Very limited preclinical data; no clinical efficacy evidence

*Note:* This table summarises the current evidence landscape based on published literature. The ‘translational stage’ is a descriptive classification based on study type, sample size and degree of clinical validation, not a formal methodological quality grading as in systematic reviews.

### 5.2 Safety Considerations

In gynaecological populations, the most frequently reported adverse effects of GLP‐1 receptor agonists are mild, self‐limiting gastrointestinal symptoms—nausea, vomiting and diarrhoea—that typically emerge during dose escalation and resolve without intervention [99]. Nevertheless, several safety domains warrant particular attention when these agents are prescribed to women of reproductive age. The use of GLP‐1 receptor agonists during pregnancy and lactation remains a key area of uncertainty. Although current evidence has not confirmed human teratogenicity, reproductive safety data are insufficient to recommend their use in gestation or breastfeeding [106]. Animal studies have demonstrated fetal growth restriction and skeletal abnormalities [99], and regulatory agencies classify these agents as pregnancy Category C, advising effective contraception during therapy together with a washout period of at least two months before conception [107, 108]. A large‐scale systematic review published in 2026 noted that early‐pregnancy exposure was not consistently associated with increased risks of major congenital malformations or adverse neonatal outcomes in adjusted analyses, yet data on continued use throughout gestation are still limited [109]. Lactation safety information is similarly sparse; one pharmacokinetic study detected no semaglutide transfer into human milk, but clinical guidelines continue to advise against use during breastfeeding because the evidence base remains inadequate. In the adolescent PCOS population, long‐term safety data are also scarce. While GLP‐1 receptor agonists have shown metabolic benefits that include weight reduction, improved insulin sensitivity and restoration of ovulatory function, their potential impact on growth, pubertal development and maturation of the hypothalamic—pituitary‐ovarian axis has not been systematically evaluated [110], and current evidence‐based guidelines for PCOS management do not yet incorporate these agents as standard therapy in adolescents [111, 112]. Beyond gastrointestinal tolerability, longer‐term safety concerns extend to other organ systems. Meta‐analytic data indicate a 32% increased risk of gallbladder disorders and a 17% increased risk of pancreatitis associated with GLP‐1 receptor agonist use [113], and preclinical studies have raised concerns about C‐cell hyperplasia and medullary thyroid carcinoma in rodent models exposed to high‐dose liraglutide; although these findings have not been replicated in non‐human primates and human data remain inconclusive [114], safety signals for pancreatitis, C‐cell hyperplasia and medullary thyroid carcinoma persist across both GLP‐1 receptor agonists and dual GLP‐1/GIP agonists and dedicated long‐term cohort studies in gynaecological populations are absent [115]. Drug–drug interactions also merit careful evaluation. When GLP‐1 receptor agonists are co‐administered with oral contraceptives or other hormonal agents, pharmacokinetic interactions—particularly during initiation or dose titration—may theoretically alter contraceptive efficacy [116], and while the risk of hypoglycemia is low with monotherapy, it increases significantly when these agents are combined with insulin or sulfonylureas. Given that concomitant use of metformin, progestins or combined oral contraceptives is common in gynaecological practice, prospective assessment of additive adverse effects and altered drug exposure profiles is warranted. In summary, although GLP‐1 receptor agonists offer considerable therapeutic promise for metabolic and reproductive health in women, their safety profile in pregnancy, adolescence, long‐term use and polypharmacy contexts remains incompletely characterised and dedicated prospective studies together with gynaecology‐specific registries are urgently needed to inform evidence‐based clinical decision‐making [115].

## 6 Conclusions

In summary, this review has systematically examined the biological characteristics and core signalling networks of incretins, with particular attention to their mechanisms of action and clinical translational potential in common gynaecological diseases. The incretin hormones, chiefly GLP‐1 and GIP, are now recognised to exert far‐reaching metabolic and hormonal regulatory functions that extend well beyond the pancreas. Through pathways such as cAMP/PKA, PI3K and AMPK, they mediate a broad spectrum of extra‐pancreatic activities, including anti‐inflammatory and immunomodulatory effects, cardiovascular protection and modulation of reproductive processes. Among gynaecological conditions, the most compelling clinical evidence has accumulated for PCOS, where GLP‐1 receptor agonists consistently improve insulin resistance, metabolic profiles, menstrual regularity and ovulatory function, especially in women with overweight or obesity. Although GIP signalling appears to participate in the pathogenesis of PCOS, the clinical data supporting its therapeutic manipulation remain limited. In contrast, the roles of incretins in endometriosis, intrauterine adhesions and gynaecological malignancies are still at a preliminary stage and rest largely on preclinical observations. Any therapeutic benefits in these disorders may be partially indirect, arising from the amelioration of systemic metabolic disturbances, while the existence of direct, tissue‐specific actions within the reproductive organs awaits rigorous validation. Collectively, incretin‐based therapies represent a promising interdisciplinary bridge between endocrinology and gynaecology, opening novel avenues for the management of metabolic–reproductive disorders.

Looking ahead, several research priorities emerge. Large‐scale, multicenter randomised controlled trials are urgently needed to establish the long‐term efficacy and safety of incretin‐based therapies in well‐characterised gynaecological populations, ideally with reproductive outcomes as primary endpoints. In parallel, the therapeutic promise of dual and multi‐target incretin agonists, such as GIP/GLP‐1 receptor co‐agonists, warrants dedicated exploration across a range of gynaecological diseases. A deeper dissection of tissue‐specific incretin signalling within the reproductive tract will be essential to disentangle direct hormonal effects from the indirect consequences of improved metabolic health. Finally, the identification of predictive biomarkers should be pursued to enable patient stratification and to realise the goal of precision therapy in this rapidly evolving field.

## Author Contributions

**Jiayu Yan:** writing – original draft, conceptualization, data curation, writing – review and editing, methodology. **Minyue Cao:** formal analysis, investigation. **Yan Ding:** methodology. **Yiqin Zhang:** validation. **Yihan Sun:** software. **Genyi Jiang:** investigation. **Yanli Zhang:** supervision. **Luyao Kang:** software. **Xue Zhou:** visualization. **Jing Luo:** writing – original draft, validation. **Bilan Li:** supervision, funding acquisition, project administration, writing – review and editing.

## Funding

This study was supported by grants from the National Natural Science Foundation of China (Grants 82372925 and 82674378), Shanghai Pujiang Program (Grant 25PJD102), Shanghai Medical Star of Tomorrow Youth Medical Talent Development Funding Program (Grant 20264Z0005), the Shanghai Shenkang Hospital Development Center (Grant 2025SKMR‐30, SHDC22026205), Shanghai Oriental Talents (Grant QNWS2024042), the Clinical Research Program of Shanghai First Maternity and Infant Hospital (Grant 2025B02).

## Conflicts of Interest

The authors declare no conflicts of interest.

## Data Availability

This article is a review of the existing literature and does not report any new primary data. All data discussed or analysed in this review are available from the original publications cited in the reference list.
